# Mechanism of Action of Bortezomib and the New Proteasome Inhibitors on Myeloma Cells and the Bone Microenvironment: Impact on Myeloma-Induced Alterations of Bone Remodeling

**DOI:** 10.1155/2015/172458

**Published:** 2015-10-22

**Authors:** Fabrizio Accardi, Denise Toscani, Marina Bolzoni, Benedetta Dalla Palma, Franco Aversa, Nicola Giuliani

**Affiliations:** ^1^Myeloma Unit, Department of Clinical and Experimental Medicine, University of Parma, 43126 Parma, Italy; ^2^UO di Ematologia e CTMO, Azienda Ospedaliero-Universitaria di Parma, 43126 Parma, Italy

## Abstract

Multiple myeloma (MM) is characterized by a high capacity to induce alterations in the bone remodeling process. The increase in osteoclastogenesis and the suppression of osteoblast formation are both involved in the pathophysiology of the bone lesions in MM. The proteasome inhibitor (PI) bortezomib is the first drug designed and approved for the treatment of MM patients by targeting the proteasome. However, recently novel PIs have been developed to overcome bortezomib resistance. Interestingly, several preclinical data indicate that the proteasome complex is involved in both osteoclast and osteoblast formation. It is also evident that bortezomib either inhibits osteoclast differentiation induced by the receptor activator of nuclear factor kappa B (NF-*κ*B) ligand (RANKL) or stimulates the osteoblast differentiation. Similarly, the new PIs including carfilzomib and ixazomib can inhibit bone resorption and stimulate the osteoblast differentiation. In a clinical setting, PIs restore the abnormal bone remodeling by normalizing the levels of bone turnover markers. In addition, a bone anabolic effect was described in responding MM patients treated with PIs, as demonstrated by the increase in the osteoblast number. This review summarizes the preclinical and clinical evidence on the effects of bortezomib and other new PIs on myeloma bone disease.

## 1. Introduction

Bone disease, the hallmark of multiple myeloma (MM), is characterized by the presence of pure lytic lesions instead of solid tumors [1, 2]. Radiological bone lesions are found to be present in about 70–80% of newly diagnosed MM patients. It has been reported that 67% of MM patients display lytic lesions and 20% osteoporosis or pathologic fractures [3]. Up to 84% of the patients were found to develop skeletal lesion during the disease [3]. Skeletal-related events (SREs) consist of pathological or vertebral fractures, hypercalcemia, severe bone pain, and need for surgery/radiotherapy that affect the MM patients by decreasing the quality of life [4]. Although conventional radiography is the standard diagnostic procedure for the detection of skeletal involvement defining the presence of lytic lesions, its utility is limited as lytic lesions can be detected only after 30% trabecular bone loss [5]. The whole-body low-dose computed tomography (CT) is a reproducible technique for defining bone disease in MM patients with higher sensitivity compared to the conventional X-ray [6]. Magnetic resonance imaging (MRI) can show increased marrow cellularity due to myeloma cell infiltration, which is extremely useful in identifying the focal lesions in the absence of evident osteolysis [7]. Positron emission tomography combined with CT (PET/CT) using an 18-F labeled deoxyglucose (FDG) is being used to identify the focal growth of the myeloma cells in the skeleton [8, 9].

Osteolytic lesions are due to a profound alteration of the unbalanced and uncoupled bone remodeling process along with an increase in the osteoclast formation and activity together with the absence of osteoblastic response [2, 10]. Nitrogenous bisphosphonates are the mainstay therapy approved for myeloma bone disease that induces osteoclast apoptosis by inhibition of mevalonate pathway, preventing SREs and reducing bone pain [11]. However, anabolic agents are not available for the treatment of myeloma bone disease. Therefore, this review aims to explore the mechanisms of action of the proteasome inhibitors (PIs), including bortezomib and other next generation PIs, with particular interest in their effects on osteoclast activity and anabolic effects on osteoblasts. The potential effect of PIs on patients with bone disease in a clinical setting will also be summarized and discussed in the paper.

## 2. Pathophysiology of Myeloma-Induced Alterations of Bone Remodeling

The interaction between myeloma cells and the bone marrow (BM) microenvironment, through vascular cell adhesion molecule-1 (VCAM-1) and *α*4*β*1 integrin, stimulates the production of several proosteoclastogenic factors, including the receptor activator of nuclear factor kappa B (NF-*κ*B) ligand (RANKL) [12]. The alteration of the RANK/RANKL pathway is the main mechanism involved in the bone destruction in MM [13, 14]. RANK is a transmembrane signaling receptor located on the surface of osteoclast precursors, whereas RANKL is expressed on BM stromal cells (BMSCs) and osteoblasts and secreted by activated lymphocytes [13, 14]. Through the NF-*κ*B and JunN terminal kinase pathways, the RANK/RANKL signal enhances the osteoclast survival by increasing the bone resorption [13, 14]. Myeloma cells can disrupt the interplay between RANKL and its soluble decoy receptor osteoprotegerin (OPG) by increasing the RANKL and decreasing the OPG expressions and promoting the formation and activation of osteoclasts [15]. Moreover, several studies have demonstrated that the levels of soluble RANKL and OPG correlated with advanced bone disease having a prognostic impact [13]. The role of RANKL/OPG pathway in bone destruction has also been confirmed in murine MM models. These models have demonstrated that RANKL, either blocked by a soluble form of RANK receptor or OPG, has inhibited the bone destruction [13, 16]. The interaction between BMSCs and myeloma cells also stimulated the activation of NF-*κ*B and p38 mitogen-activated protein kinase (MAPK) pathways. Specifically, the inhibition of p38 decreased the adhesion of myeloma cells to BMSCs, reduced the myeloma cell proliferation, and shortened the tumor burden in the murine MM model [17, 18]. Chemokine (C-C motif) ligand 3 (CCL3), being an RANKL independent inducer of osteoclast formation, can enhance both RANKL and interleukin- (IL-) 6 stimulated osteoclast formation [19]. The level of CCL3, produced directly by the human myeloma cells, correlates with the osteolytic bone lesions in MM patients [20, 21]. Moreover, either an antisense sequence anti-CCL3 or a neutralizing antibody against CCL3 reduces the bone destruction in mouse MM models [21]. IL-3 and IL-7 are also involved in osteoclastic bone resorption in MM [1, 22]. Moreover, Activin A, a member of TGF-*β* family, has been identified as a factor involved in IL-3 induced osteoclast activation in MM patients [23, 24].

Along with increased bone resorption, myeloma bone disease is characterized by suppressed osteoblast activity. MM patients show lower levels of bone formation markers, such as alkaline phosphatase (ALP) and osteocalcin (OC), and increased bone resorption markers [25]. Osteoblast suppression occurs mainly due to the blockage of the osteoblast differentiation from progenitors into the BM. The osteogenic differentiation of stromal cells requires the activity of the runt-related transcription factor 2 (Runx2/Cbfa1) [26]. The role of Runx2 in MM-induced osteoblast inhibition has been demonstrated in coculture systems performed between myeloma cells and osteoprogenitor cells [27]. Myeloma cells can inhibit osteoblast differentiation by reducing the number of both the early and late osteoblast precursors and decreasing the expression of ALP, OC, and type I collagen [27]. MM-induced Runx2 inhibition in the osteoprogenitor cells is mediated by the cell-to-cell contact between myeloma and osteoprogenitor cells [27]. Moreover, it has been reported that the MM patients had increased levels of transcriptional repressor Gfi1 compared with controls and that Gfi1 was a novel transcriptional repressor of Runx2 [28, 29]. IL-7 is involved in the Runx2 inhibition in osteoblast progenitors and in the consequent suppression of the osteoblast formation [27, 30]. Tumor necrosis factor- (TNF-) *α* is an inflammatory cytokine increased in MM and BM microenvironment that block osteogenic differentiation by suppressing the Runx2 and osterix expressions [31, 32]. Consistently, both anti-IL-7 and anti-TNF-*α* antibodies blocked the Gfi1 upregulation in BMSCs [28]. IL-3 has a dual role in myeloma bone disease; apart from stimulating the bone resorption, IL-3 can also inhibit the differentiation of preosteoblast at concentrations similar to those seen in BM plasma from MM patients [22, 33]. The inhibitors of the canonical wingless-type (Wnt) signaling, such as soluble frizzled-related proteins, sFRP-2, sFRP-3, and Dickkopf-1 (Dkk-1) [34–38], are involved in the pathogenesis of myeloma bone disease. The canonical Wnt signaling, through binding of Wnt proteins to the frizzled receptor and low-density lipoprotein receptor-related protein (LRP-5/6) coreceptor, leads to the translocation of *β*-catenin to the nucleus. Here, it interacts with members of the T-cell factor (TCF)/lymphoid enhancer factor (LEF) family in order to activate the osteoblast transcription factors and osteoblast formation [39]. Previous literature data indicate that the deregulation of canonical Wnt signaling in myeloma cells causing overexpression of Dkk-1 or frizzled-related protein gene FRZB is associated with a high incidence of bone lesions in MM patients [36, 37]. Moreover, higher Dkk-1 levels in BM correlate with the presence of focal bone lesions in MM patients [37].

Besides negative regulation of osteoblast differentiation, myeloma cells may affect osteoblast proliferation and induce osteoblast apoptosis in coculture systems by sensitizing cell death mediated by TRAIL [40–42]. In the last few years, studies have focused on the role of osteocytes, the terminally differentiated cells derived from osteoblasts, to partially regulate bone remodeling through cell death [43, 44]. Recently, studies have reported an increase of osteocyte death in MM patients bone disease in relation to the presence of bone lesions and the number of osteoclasts [45]. These data, which were confirmed by ultrastructural* in vitro* analysis on coculture system, showed that myeloma cells can induce cell death in human preosteocytes, [45] which also regulate the osteoclast activities. In particular, living osteocytes produce soluble factors that inhibit osteoclast formation, whereas the apoptotic or autophagic osteocytes lose this inhibitory effect and promote bone resorption [46]. Indeed, apoptotic bodies produced from the osteocyte-like cells support osteoclastogenesis [46].

## 3. The Proteasome Complex and Its Inhibition

The proteasome, a multicatalytic enzyme complex located in the cytoplasm and cell nucleus, is involved in the adenosine triphosphate- (ATP-) dependent intracellular proteolysis by ensuring the rapid degradation of the target proteins with a chain of ubiquitin [47]. The ubiquitin-proteasome pathway (UPP) is the principal pathway by which the cellular proteins, such as the proteins involved in cell cycle, transcription, DNA repair, and apoptosis, are degraded [47, 48]. The control of the timed protein degradation is essential for controlling the intracellular protein levels and the cellular function [47–50]. The 26S proteasome is formed by 20S proteolytic core region and 19S regulatory particle [47–50]. The 20S core region is made up of 28 subunits arranged in four stacked heptameric rings to form a chamber where the proteolysis can occur [51]. The two outer and inner rings are composed of 7*α* and 7*β* different subunits, respectively, arranged one above the other as *α*-*β*-*β*-*α* [51]. Degradation of a protein involves coupling of a polyubiquitin chain through the action of three enzymes in an ATP-dependent manner [49, 51, 52]. This polyubiquitin chain acts as a flag to target the protein for degradation. When the ubiquitin molecules are removed, the protein is transferred into the inner catalytic chamber of the 20S proteasome where three different catalytic activities cleave the ubiquitinated protein into small peptides [52, 53]. The catalytic activities, linked to two central *β*-rings, are classified into three categories: chymotrypsin-like (CT-L), trypsin-like (T-L), and caspase-like (C-L) activities [51, 53]. Since UPP is involved in essential biological processes, the malfunction in this pathway is associated with a variety of diseases leading to the development of PIs. The malignant cells are more sensible to the inhibition of proteasome compared to the normal cells due to their high proliferation and protein synthesis rate. In particular, the clonal myeloma plasma cells secret high amount of immunoglobulin (Ig) which are generally transported out of endoplasmic reticulum through the unfolded protein response (UPR) pathway, for proteasomal degradation [54–56]. However, if the stress is prolonged and severe as caused by PIs, the UPR pathway leads to cell cycle arrest and apoptosis [54, 57, 58]. Thus, the proteasome inhibition occurring in MM patients is sufficient to kill the malignant plasma cells but not the normal cells [59, 60]. One of the first mechanisms attributed to PIs was the inhibition of the transcription factor NF-*κ*B activity. It is well known that NF-*κ*B plays an important role in promoting growth, survival, and chemoresistance of myeloma cells in BM through the regulation of IL-6 and insulin-like growth factor 1 (IGF-1) expression [61, 62]. Moreover, it regulates various tumor-related processes such as induction of angiogenesis and suppression of apoptosis [61, 63]. Inhibition of proteasome activity prevents degradation of the NF-*κ*B inhibitor I-*κ*B, which blocks the binding of NF-*κ*B to the promoters of the target genes such as antiapoptotic genes and IL-6 [63, 64].

## 4. Proteasome Inhibition and Bone Microenvironment Cells

Proteasome inhibition is involved in bone remodeling. As described above, the binding of RANKL to RANK on the surface of osteoclast precursors activates NF-*κ*B that promotes the osteoclast maturation and bone resorption [13, 14]. Thus, the proteasome-dependent inhibition of NF-*κ*B leads to a reduction in the RANKL-mediated osteoclast differentiation. Moreover, it has been demonstrated that the PIs, MG-132 and MG-262, inhibit both osteoclast formation and resorption capacity, and this correlates with the extent of NF-*κ*B binding capacity [65, 66].

On the other hand, the proteasome pathway also regulates the bone formation. It has been shown in an MM mouse model that treatment with PIs resulted in an increase in the bone mineral density and a concomitant reduction in the osteoclast numbers [67–69]. The compounds that inhibit proteasome activity, such as lactacystin and epoxomicin, stimulate bone formation in a dose-dependent manner affecting the increased expression of bone morphogenetic protein- (BMP-) 2 by osteoblasts [69]. This impact suggests that PIs and the proteasome pathway may have a role in bone remodeling.

**Figure 1:**
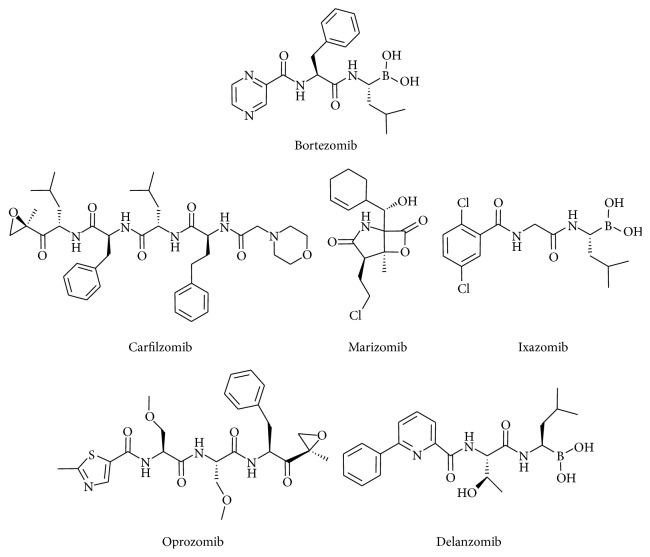
Bortezomib and the new PIs. Chemical structure of bortezomib and the new PIs. PIs: proteasome inhibitors.

Bortezomib, also known as PS-341, is the first class of PIs approved for treatment of MM [70–72]. Chemically, it is a dipeptidyl boronic acid that binds reversibly to CT-L subunit of the proteasome [73, 74] (Figure 1). It has also been reported to bind to C-L and T-L subunits with lower affinity [73, 74]. Although bortezomib is a reversible inhibitor, the boronate-proteasome complex has a low degree of dissociation and remains stable for several hours [74].

An increasing number of studies focused on the role of bortezomib in MM-related bone disease. It has been demonstrated that bortezomib affects RANKL-induced osteoclast differentiation in a dose-dependent manner in both the early and late stages through the modulation of p38, activator protein-1 (AP-1), and NF-*κ*B pathways [65, 66]. The SCID-rab mice bearing myeloma, additionally, showed a reduction in the osteoclast number after the bortezomib treatment [75].

Bortezomib not only inhibits the osteoclast function but also affects the osteoblast differentiation. In preclinical models, it has been reported that bortezomib can induce osteoblast phenotype in human mesenchymal stromal cells (MSC) without affecting the number of osteoblast progenitors and viability of mature osteoblasts [76]. The* in vitro* effect was associated with an increase in both the Runx2 activity and expression of osteoblast markers such as type I collagen, without affecting the canonical Wnt signaling [76]. These* in vitro* observations also confirmed the bone biopsies of MM patients treated with bortezomib showing that the responding patients had more osteoblastic and Runx2-positive cells compared to the control groups [76]. The bone anabolic effect of bortezomib relies on the activation of *β*-catenin/TCF signaling. This effect shows that bortezomib promotes matrix mineralization by osteoprogenitor cells through the stabilization of *β*-catenin and induction of TCF transcriptional activity [77]. It has been demonstrated that bortezomib can enhance the differentiation of murine MSCs towards osteoblasts, rather than the more differentiated osteoblast progenitors [78]. Moreover, in both the mouse models implanted with MSCs and osteoporosis, the treatment with low doses of bortezomib resulted in an increase in the bone formation. No effect on the osteoclast activation and differentiation generated from murine BM mononuclear cells was observed [78]. It was also demonstrated that bortezomib stabilizes Runx2 activity consistently with the previous studies concluding that PIs should prevent Runx2 degradation [69]. Further, bortezomib and other PIs also stimulate the bone formation in mouse calvarial organ culture by increasing the BMP-2 production. This is positively correlated with their ability to inhibit the proteasome activity [69, 79]. One of the possible mechanisms in which the PIs stimulate BMP-2 expression involved the protein Gli-3. Gli-3 is degraded in a proteasome-dependent manner and its truncated form is a potential inhibitor of BMP-2 transcription. Its overexpression in osteoblast precursors has been reported to inhibit the effects of PIs on BMP-2 expression. The PIs are also able to prevent the proteolytic processing of Gli-3, the generation of its truncated form, and the suppression of BMP-2 gene transcription [69]. On the other hand, in another study, bortezomib was found to increase the expression of ALP and OC in mesenchymal cell line with an effect similar to BMP-2, but without affecting the BMP-2 target gene expression [80]. Bortezomib inhibits Dkk-1 gene expression and protein level in both mice treated with* calvariae* and BMSCs, which also suggested its ability to modulate the canonical Wnt signaling [79]. Using the severe combined immunodeficiency- (SCID-) rab mouse as a model, it has been reported that the mice responding to bortezomib showed a significant increase in both BMD and osteoblast and decrease in osteoclast numbers [67]. The increased BMD is not seen in responsive melphalan-treated mice, suggesting that the effect of bortezomib on bone is not only due to the tumor burden reduction. A histomorphometric analysis revealed that the myelomatous bones from bortezomib-treated hosts showed increased trabecular thickness and trabecular numbers associated with a higher number of osteoblasts and a lower number of osteoclasts in comparison to the control groups [67]. Osteoblasts and MSCs express the vitamin D receptor (VDR), and the effects of vitamin D on osteogenic differentiation have been demonstrated both* in vitro* and in mouse models [81, 82]. Recently, it has been showed that the simultaneous treatment with bortezomib and vitamin D strongly stimulated the VDR signaling and increased the vitamin D-dependent expression of osteoblastic differentiation markers, such as OC and osteopontin, by both the human MSCs and osteoblasts. Bortezomib also blunts the downregulation of OC and osteopontin, induced by coculture with myeloma cells [83]. Moreover, the stimulatory effect of bortezomib on VDR signaling may be due to the decreased proteasomal degradation of the VDR [83].

Recently,* in vitro* data indicated that the bortezomib or MG262 treatment for 12–24 hours would significantly blunt the osteocyte cell death induced by the myeloma cells. In addition, treatment with PIs reduced the high doses of dexamethasone-induced death of MLO-Y4. Parathyroid hormone (PTH) short-term treatment also potentiated the* in vitro* effects of bortezomib and MG262 on the dexamethasone-induced death of osteocytes [84]. The data also indicated that the anabolic effects of bortezomib and PIs may have been mediated by their impact on the osteocytes rather than on osteoblasts.

**Figure 2:**
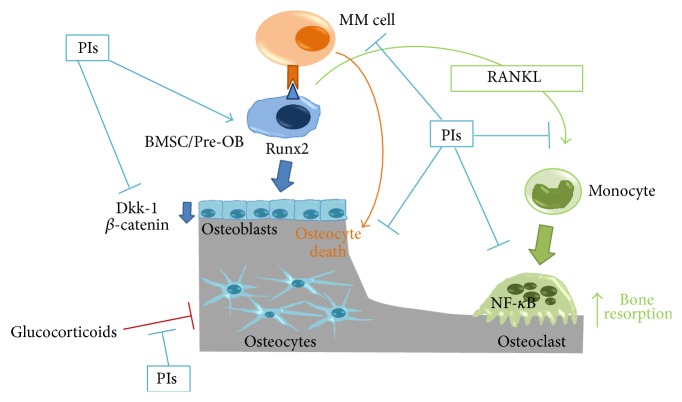
Effects of PIs on osteoblast and osteoclast remodeling in MM. PIs directly act on myeloma cells and on MM-induced alterations of bone remodeling. PIs block osteoclast formation from monocyte and the effects of RANKL on osteoclastogenesis. A direct effect of PIs on mature osteoclast has been shown. PIs stimulate osteogenic differentiation of BMSCs and osteoblast progenitors increasing osteoblast number and function. A stimulatory effect of PIs on the osteogenic transcription factor Runx2 has been demonstrated. PIs reduce Dkk-1 production and consequently affect *β*-catenin. PIs stimulate osteocyte viability and blunt the effect of glucocorticoid on osteocytes. PIs: proteasome inhibitors; RANKL: receptor activator of nuclear factor kappa B (NF-*κ*B) ligand; BMSC: bone marrow stromal cell; Pre-OB: osteoblast progenitor.

Thus, several mechanisms underlying the effects of PIs and bortezomib on bone remodeling demonstrate that these drugs inhibit osteoclast formation and activity with a significant anabolic effect (Figure 2).

## 5. Second Generation of PIs and Their Possible Effects on Bone Remodeling

Recently, novel PIs have been developed to overcome bortezomib resistance. The second generation of PIs, such as carfilzomib, marizomib, ixazomib, oprozomib, and delanzomib, differed in the chemical structure, biological properties, and mechanisms of action [85] (Figure 1).

*Carfilzomib* (PR-171) is a tetrapeptide epoxyketone analog of epoxomicin, an epoxyketone family member of natural PIs [86, 87]. It binds irreversibly to CT-L catalytic subunits of proteasome so that the reestablishment of proteasome function is possible only by the synthesis of new single subunits [88, 89]. In high doses, it also inhibits the T-L and C-L activities [87]. In contrast to bortezomib, which binds with different serine proteases contributing to some of the neurotoxicity, carfilzomib binds irreversibly with proteasome only and not with other proteases [86–89]. Preclinical studies have demonstrated that the greater selectivity of carfilzomib for the CT-L, compared to bortezomib, revealed little off-target activity and dose flexibility in the xenograft models [87–89].

Recently, it has been demonstrated that carfilzomib stimulates,* in vitro*, MSCs differentiation into bone-forming osteoblasts by increasing the matrix mineralization and calcium deposition [68, 90, 91]. Osteoblasts derived from MM-MSC patients, treated with clinically relevant doses of carfilzomib, showed an increase in the ALP activities [68]. Carfilzomib inhibits osteoclast differentiation and function at cytotoxic concentrations to myeloma cells without affecting the precursor viability. This effect seems to be due to the disruption of RANKL-induced NF-*κ*B signaling and the reduced *α*V*β*3 integrin expression involved in bone resorption activities of osteoclasts [68]. During the osteoblast differentiation, carfilzomib reduced RANKL expression by inhibiting their ability to stimulate osteoclastogenesis. The* in vitro* evidences were confirmed by the* in vivo* studies on both the nontumor bearing mice and 5TMG1 model, which suggested that the potential efficacy of the treatment in other pathological disorders is characterized by bone disease [68]. The molecular mechanisms by which carfilzomib promotes MSC differentiation are still under investigation. It has been reported that *β*-catenin/TCF pathway is involved in regulating the MSCs and osteoblasts differentiation [39]. Carfilzomib also induces the Wnt-independent nuclear accumulation of active *β*-catenin as well as the activation of the transcription factor TCF in both osteoblastic-like cell and stromal cell lines in the MM-MSC patients [90]. In the last years, several authors have shown that Notch1 pathway regulates the osteogenic differentiation by suppressing the Runx2 activity in BM mesenchymal progenitors [91–93]. Moreover, the induction of osteogenic differentiation suppresses the Notch1 activity. Recently, it has been demonstrated that the carfilzomib-induced stimulation of osteogenesis is associated with Notch1 signaling inhibition [91]. The role of carfilzomib in PTH signaling is to inhibit the PTH-induced* RANKL* mRNA expression by blocking the histone deacetylase 4 (HDAC4) proteasomal degradation in osteoblasts [94]. However, carfilzomib fails to affect the PTH-dependent inhibition of OPG. Using coculture system between osteoblastic cell line and osteoclast precursors cells, it has been shown that high concentrations of carfilzomib can inhibit PTH-induced osteoclast formation and activity. This inhibition decreases the NF-*κ*B activation without affecting the cell viability [94].

*Marizomib* (NPI-0052) is the first natural PI included in the MM clinical research [95, 96]. It is an orally bioactive *β*-lactone derived from obligate marine bacteria actinomycetes,* Salinispora tropica*, and is structurally different from bortezomib and carfilzomib [96]. Marizomib inhibits all the enzymatic activities of proteasome binding with high affinity to the CT-L and T-L catalytic sites and lower affinity to the C-L site [96]. Similarly to bortezomib, marizomib also inhibits the canonical NF-*κ*B pathway and secretion of IL-6, TNF-*α*, and IL-1*β* but at lower concentrations than bortezomib [97, 98]. Bortezomib requires caspase-8 and caspase-9, whereas marizomib induces the apoptotic effect mainly through caspase-8 signaling that allows it to overcome the resistance of myeloma cells conferred by Bcl-2 mutations [97, 98]. The overexpression of Bcl-2 is demonstrated to protect the myeloma cells by bortezomib and to some extent by marizomib too, due to its caspase-9 activation [98]. The marizomib potentiated apoptosis is induced by TNF-*α*, bortezomib, and thalidomide with a concomitant downregulation of cell proliferation and survival proteins (such as cyclin D1, c-Myc, Bcl-2, Bcl-xl, and survivin). The protein involved in migration and angiogenesis, such as matrix metalloproteinase (MMP-9) and vascular endothelial growth factor (VEGF), also induces the apoptosis [99]. Marizomib did not affect the viability of BMSCs, rather blocked the production of IL-6 that is triggered by myeloma cells and BMSC interaction. It also induced apoptosis in myeloma cells in the presence of IL-6 and IGF-1 [98, 99]. The potent antitumor activity of marizomib has been confirmed* in vivo* studies on a human plasmacytoma xenograft mouse model [98].

A few studies have focused on the marizomib's effect on osteoclastogenesis and osteoblastogenesis. Nevertheless, it was well demonstrated that carfilzomib inhibits RANKL-induced osteoclastogenesis without affecting the viability of osteoclast-like cells [99].

*Ixazomib* (MLN9708), an analog of boronic acid, is orally administered with greater potential activity against myeloma cells than bortezomib [96, 100]. In comparison to bortezomib, ixazomib hydrolyzes immediately in the aqueous solution or plasma to its biologically active form MLN2238, displaying shorter half-life and wider distribution in blood [100–102]. It inhibits not only the CT-L subunit in higher concentrations, but also the C-L and T-L subunits of the proteasome. Ixazomib showed a shorter proteasome dissociation half-life than bortezomib by improving its pharmacokinetic and pharmacodynamic profile [100–103]. The antitumor activity of ixazomib depends on the activation of caspase-8, caspase-9, and caspase-3 and the upregulation of p53 and p21. Treatment with ixazomib upregulates the transcription factors that respond to the endoplasmic reticulum stress. It also inhibits both the canonical and noncanonical NF-*κ*B signaling in myeloma cells by reducing the BMSCs-induced proliferation of myeloma cells [96].

Ixazomib inhibits* in vitro* osteoclastogenesis and osteoclast resorption. These effects involved the F-actin ring damage, blockade of the NF-*κ*B activation induced by the RANKL, and downregulation of *α*V*β*3 integrin. Ixazomib also promotes* in vitro* osteoblastogenesis and osteoblast activity, at least in part, by the activation of *β*-catenin/TCF signaling. It also encourages the upregulation of the inositol-requiring enzyme 1 (IRE1) component of the unfolded protein response. Finally, ixazomib demonstrates significantly reducing the bone disease in MM mouse model [100].

*Oprozomib* (ONX0912) is a novel orally administered epoxyketone that is derived from carfilzomib [104]. It binds irreversibly to CT-L subunit of proteasome, resulting in longer duration of inhibition compared to bortezomib [104]. In an* in vitro* study, it has been demonstrated that oprozomib inhibits growth and migration of myeloma cell lines and induces apoptosis through the activation of caspase-8, caspase-9 and caspase-3 and poly(ADP) ribose polymerase (PARP) [104].

Compared to carfilzomib, oprozomib inhibits osteoclast differentiation and functions without affecting the osteoclast precursor viability. The treatment of osteoclast precursors with oprozomib inhibited RANKL-induced NF-*κ*B activation caused by the damaged proteasomal degradation of I-*κ*B. Similarly, oprozomib promotes osteogenic differentiation* in vitro* increasing the ALP activity and other osteogenic markers that modulate the transforming growth factor *β* (TGF *β*), MAPK, and UPR pathway [68]. Both the antitumor and anabolic effects of oprozomib were confirmed in an* in vivo* mouse model [68].

*Delanzomib* (CEP-18770) is a boronic acid-based PI that inhibits, reversibly, CT-L and C-L activities [105]. Delanzomib has been demonstrated to decrease the NF-*κ*B activity and induce apoptosis in myeloma cells with a good cytotoxic profile towards normal cells [105, 106]. In the preclinical mouse model of MM, it has been demonstrated that delanzomib can efficiently induce an improved response against bortezomib-resistant cells [106]. During the culture, delanzomib showed antiangiogenic and antiosteoclastogenic activities [105]. In the preclinical studies, delanzomib showed an enhanced anti-MM activity of bortezomib and melphalan, and it also reduced the tumor growth in combination with dexamethasone and lenalidomide [107, 108].

## 6. Effects of PIs Treatment on Bone Disease in MM Patients

The original observation by Zangari et al. [109] on ALP increase in a 63-year-old woman affected by relapse MM responding to bortezomib encouraged more large-scale analysis in three data sets from clinical trials. This confirmed a correlation between ALP increase and its response to bortezomib therapy [109]. Retrospective analysis of ALP variation in SUMMIT and APEX trials displayed a statistically significant difference in the median levels of ALP in responders to bortezomib versus nonresponders maximum in the eighth and sixth week, respectively [110–112]. In the APEX trial, considering only the responding patients of both the groups, median ALP variation was higher in bortezomib group in comparison to the dexamethasone group. This observation suggests that both the direct and indirect effects on the bone disease occurred during the bortezomib treatment [112]. Similarly, a recent retrospective analysis of 67 relapse or refractory MM patients who were treated with carfilzomib demonstrated that elevation in ALP levels correlated with the response to the treatment [113].

Several studies after the APEX trial analysis confirmed the positive effects of PIs on bone formation and resorption markers [114–121].

Biochemical markers of the bone remodeling represent an important tool to check the alterations in the bone turnover that occurs in MM patients with extensive bone disease. They are particularly useful in evaluating the response to the antiresorptive or anti-MM therapy with a significant impact on the bone turnover. Bone resorption markers are known to include collagen N-terminal cross-linking telopeptide of type I collagen (NTX), C-terminal cross-linking telopeptide of type I collagen (CTX), and I-terminal cross-linking telopeptide of type I collagen (ICTP) that represent bone-specific products of osteoclast-mediated degradation of triple-helix collagen. Tartrate-resistant acid phosphatase isoform-5b (TRACP-5b) is an osteoclast-specific serum enzyme that reflects the total osteoclastic number and activity. Bone formation markers include procollagen type I N-propeptide (PINP) and procollagen type I C-propeptide (PINC) derived from degradation of procollagen during the deposition of bone matrix. Bone-specific ALP (bALP) and OC are well-known indicators of osteoblast bone formation and activity [122].

Terpos et al. [119] showed an increase in bALP and OC in the relapse MM patients treated with twice-weekly bortezomib for four cycles. The change in bALP was marked in responders versus nonresponders and correlated significantly with the type of response. Dkk-1 levels at baseline were increased both in the study population compared to the control groups and in the MM patients with high of bone disease compared to all other groups [119]. After four cycles of bortezomib, Dkk-1 serum levels decreased significantly compared to the baseline, irrespective of the response to the treatment. Markers of bone resorption TRACP-5b and CTX and osteoclast regulator soluble RANKL (sRANKL) were significantly reduced after the treatment [119]. In another study, serum CTX and urinary NTX were evaluated before and after three days of each bortezomib administration performed on three MM patients [123]. Bortezomib induced a significant reduction percentage after two days compared to that in the baseline with a trend of increment after three days [123]. Lund et al. [117] assessed that the variations in bone turnover markers included bALP, PINP, Dkk-1, and NTX-I in the bisphosphonate-naïve and untreated MM patients. All patients received four cycles of twice-weekly bortezomib, initially as monotherapy and then combined with dexamethasone from the second to the fourth cycle. In the responders, bone formation markers bALP and PINP increased to the maximum value on day 42 [117]. A temporary decrease of PINP was also observed every time dexamethasone was added. Dkk-1 and NTX levels decreased to 25% and 50% in the responding patients, respectively. No changes in the bone remodeling markers were detected in nonresponders, except for a little decrease in NTX [117].

A post hoc analysis of phase III VISTA was conducted to assess the clinical skeletal events and the serum modifications in ALP and Dkk-1 during the treatment. The untreated MM patients, not eligible for transplantation, were randomized to bortezomib-melphalan-prednisone (VMP) or melphalan-prednisone (MP) alone. Bisphosphonate therapy was allowed during the treatment and follow-up period. The increase in maximum median ALP from baseline to any time point was higher by response in VMP group versus MP group, both in patients achieving CR and PR [118]. It was also noted that a statistically significant Dkk-1 reduction in serum from baseline to the day 4 of the first cycle showed opposite results to the increase in the MP subgroup. Six out of 11 patients in the VMP arm were assessed by skeletal imaging (X-ray or CT) both before and after baseline, and they showed signs of bone sclerosis suggesting an initial process of bone healing, but none was observed in MP arm [118].

In a multicenter prospective study, the primary endpoint was the bone markers variation recorded before and after four cycles of twice-weekly bortezomib in association with other agents in relapse MM patients [120]. A reduction in the Dkk-1 levels was recorded after bortezomib treatment, and the levels of OC and bALP were also found to decrease both in the responders and in nonresponders. Remarkably, the same bone markers variation was not significant in the patient group without steroid combination, which confirms the detrimental role of steroids on bone neoformation to overcome the bortezomib positive effect on osteoblast function.

Recently, a prospective study was conducted to compare the bone markers changes in 99 relapse MM patients treated with drugs combinations of lenalidomide-dexamethasone (LD) or bortezomib-lenalidomide-dexamethasone (VRD) [121]. In the VRD arm, a marked increase in bALP and OC and a reduction in sRANKL/OPG, Dkk-1, and CTX were observed after the third and sixth cycle, irrespective of the response to the treatment. RD arm patients showed an increase of Dkk-1 after six months of therapy and a significant reduction of CTX levels in responders as compared to the nonresponding patients without any other significant alterations on bone biomarkers. Additionally, two refractory patients in RD subgroup developed SREs but none in VRD. This study supports the positive bortezomib role in enhancing the bone formation and preventing bone resorption while the lenalidomide alone retains a minor effect on the bone resorption.

In addition to the studies on the markers of bone turnover, a histomorphometric study was conducted by Giuliani et al. [76] on the BM biopsies of 21 MM patients before and after the sixth to eighth cycles of twice-weekly bortezomib. This study, for the first time, displayed a significant increase in the number of osteoblastic cells/mm^2^ of bone tissue in MM patients responding to the bortezomib treatment but not in the nonresponders. Immunohistochemical staining observed a significant increase in the number of Runx2-positive osteoblastic cells in the responding MM patients compared to the nonresponders [76]. This study clearly consolidates the notion of the anabolic effect of bortezomib treatment in the MM patients.

The positive anabolic effect of bortezomib on bone healing and new matrix deposition has also been investigated by bone imaging techniques [118, 124–128]. The BMD was evaluated by dual-energy X-ray absorptiometry (DEXA) after the completion of eight cycles of twice-weekly bortezomib-dexamethasone therapy and bisphosphonates used in the 27 relapse MM patients. A total of 66% of the patients had lytic lesions in less than three areas, and 51% had osteoporosis at baseline DEXA. A significant increase in BMD was detected in the axial skeleton (L2–L4) and not in the appendicular skeleton (femoral bones). The BMD improvement correlated with the reduction of urinary NTX and increase in the serums bALP and OC [124]. Zangari et al. [125] assessed BMD changes by DEXA in 13 smoldering MM patients treated with weekly low-dose bortezomib (0,7 mg/m^2^) for nine cycles. They showed an improvement in the T-score of hip and lumbar spine at the end of the treatment in sixth and third cycle, respectively. In a case report, the effect of bortezomib as a single agent or in combination with other drugs on myeloma bone lesions was assessed using technetium-99m- (99mTc-) methyl-diphosphonate (MDP) bone scans in two MM patients.

Tc-99m MDP bone scans after the treatment revealed multiple densities with an increase in the uptake of the radiotracer on bone surfaces that is consistent throughout the new bone deposition [126]. Bone structure and remodeling alterations were also assessed by bone markers, micro-CT, bone histomorphometry, and tetracycline labeling in 16 relapse MM patients treated with twice-weekly bortezomib as a single agent. Serums bALP and OC increased considerably in the responding patients after the first cycle. In addition to bALP and OC, the increase in PTH levels was observed in the responders on day 11. Micro-CT measurements on biopsy specimens obtained on the baseline and at the end of the study showed an increase in the bone volume/total volume (BV/TV) and trabecular thickness (TbTh) after 12 doses of bortezomib and tetracycline incorporation in 63% of the analyzed biopsy samples [127]. A recent study evaluated the frequency, extent, and the patterns of BM sclerosis detected by whole-body reduced-dose CT in 79 MM patients. CT examinations were performed at baseline, during therapy, at the end, and 12 months after the termination of bortezomib treatment. Sclerosis was found to develop in 14 patients, either focal or diffuse. The mean time for the detection of skeletal sclerosis was eight months. In six patients, the mean size reduction of lytic lesions was >40%. Two patients, who were evaluated after one year from bortezomib discontinuation due to the absence of subsequent specific therapy, showed a size decrement of 17% and 100%, respectively. A considerable sclerotic modification in cancellous bone was seen in patients having no evaluable lytic bone lesions at baseline evaluation [128]. These clinical evidences further confirmed that bortezomib treatment may induce the bone healing in MM patients.

## 7. Conclusions

Osteolysis is the hallmark of MM. Bortezomib and the new PIs, which are currently being investigated in clinical trials, can affect bone remodeling. Osteoclastic formation and activity are inhibited by PIs, mainly through the blockade of RANKL signaling pathway in the osteoclast progenitors. However, the more significant impact of the bone remodeling by this class of drugs is the capacity to stimulate either the osteogenic differentiation of MSC or the osteoblastic function, leading to the consequent bone formation with a considerable anabolic effect. Osteocytes are also possible targets of PIs with a stimulatory effect on their viability. The preclinical evidence, thus, is confirmed in MM patients treated with bortezomib and more recently with carfilzomib. An improvement of the bone remodeling markers was observed in the patients treated with PIs. The histomorphometric data in MM patients treated with bortezomib prominently indicated that PIs can stimulate the bone formation process and induce the bone regeneration process. Bone healing, as well as an increase in the BMD, has also been reported in some of the patients treated with bortezomib. Overall, the literature data support the use of these drugs to restore bone integrity in MM patients.
